# Mobile Telephone Follow-Up to Ascertain Birth Outcomes in The Gambia

**DOI:** 10.1089/tmj.2019.0119

**Published:** 2020-11-05

**Authors:** Susan Laing, Karin Remmelzwaal, Max Cooper, James N'Dow

**Affiliations:** ^1^ Department of Global Health and Infection, Brighton and Sussex Medical School, Falmer, Brighton, United Kingdom.; ^2^ Department of Primary Care and Public Health, Brighton and Sussex Medical School, Falmer, Brighton, United Kingdom.; ^3^ Academic Urology Unit, University of Aberdeen, Aberdeen, United Kingdom.

**Keywords:** telehealth, mobile telephone, birth outcome, stillbirth, early neonatal death, cohort, Gambia

## Abstract

***Introduction:*** In the past decade, mobile telephone use has surged in sub-Saharan Africa, creating new opportunities in health care. Mobile telephone interventions have been used in controlled trials to improve perinatal care, but in this first cohort study of birth outcomes from The Gambia, we report the value of mobile telephone follow-up.

***Methods:*** Between December 2012 and November 2015, 1,611 women entered the cohort at their first antenatal visit to be followed through pregnancy and beyond. Potential risk factors for adverse birth outcomes were measured throughout the pregnancy. As many women left the health center within a few hours of delivery, delivered elsewhere, or failed to attend the postnatal clinics, mobile telephone follow-up was used to identify stillbirths and neonatal deaths at 7 and 28 days.

***Results:*** The immediate birth outcome was known for 968 women who delivered at the health center (60.1%). The known outcomes at birth improved from 60.1% to 85.2% following telephone calls to women who delivered elsewhere. The known outcomes at 7 days improved from 43.6% to 82.5%, and the known outcomes at 28 days improved from 32.8% to 71.5% following a telephone call.

***Conclusions:*** Previous cohort studies of birth outcomes in sub-Saharan Africa have not followed the mothers and babies after leaving the birth facility. This cohort is the first to record birth outcomes up to 28 days after the birth. Mobile telephone communications have made an invaluable contribution in intervention studies. This study has shown that mobile telephone follow-up is also an important tool in an observational study.

## Introduction

Landline telephones remain scarce in Africa, but since the early years of this century, mobile telephone ownership has surged exponentially.^1^ This remarkable expansion, now reaching into the most rural areas of sub-Saharan Africa, has eclipsed the landline stage of development and transformed communications throughout the continent. In turn, this has enabled improved access across many sectors, for example, banking, entertainment, travel, and social media. Health care delivery, however, has long been hampered by poor communications and has been relatively slow to embrace the changes that mobile communications can offer, but recently, the increasing availability of mobile phones has enabled innovative interventions in many areas of health care and given rise to a new field of medicine “mobile health.”

The past 15 years have coincided with the very rapid growth of telecommunications in The Gambia and throughout sub-Saharan Africa.^1^ In rural communities, the use of mobile telephones has proved invaluable in facilitating communications between rural health workers and more central health centers. Text messages have also been used to encourage and provide direct patient care, for example, to improve clinic attendance,^2^ improve adherence to treatment,^3^ or just to increase health knowledge.^4^ Although the participants are usually happy to receive text messages, studies that have examined the value of one-way text messages have reported mixed results,^4,5^ and in terms of results, there may be either minimal or no improvement at all. Mobile telephone calls, rather than text messages, were used in Kenya to contact the carers of children who had defaulted on immunization and to determine the reason for defaulting. The defaulting rates decreased significantly,^6^ suggesting that two-way telephone communication may be more successful than a one-way text message in achieving the result.

A systematic review of more direct telephone interventions to improve antenatal and neonatal care in low- and middle-income countries, published in 2016, suggests that there are considerable opportunities in this field.^7^ One of the largest of the randomized controlled trials, reported from Tanzania in 2012 and 2014,^8–10^ evaluated the association between mobile phone interventions (both text and telephone calls) and maternal and perinatal outcomes. In total, 2,550 pregnant women from 24 primary health care facilities were recruited at their first antenatal visit. In the intervention facilities, antenatal visits and delivery with a trained birth attendant were significantly improved and the stillbirth rate fell. Children of women in the intervention facilities had a 50% reduction in perinatal mortality.^8–10^

There are very few reports of the merits of telephone calls being used to measure the outcome of a study *per se*, rather than the two-way telephone call or one-way text being the intervention itself. In Ethiopia, a cohort of 1,059 women with standard treatments for cervical cancer were followed for up to 3 years, and telephone follow-up, where possible, was used to identify patient status following treatment.^11^ Telephone calls were also used in the postdischarge surveillance of wound infections in a cohort of 1,387 patients in Sudan,^12^ and the feasibility and lower cost of telephone follow-up versus a home visit were demonstrated in a study to monitor adverse outcomes related to antimalarial drugs in Ghana.^13^

In previous cohorts of birth outcomes reported from sub-Saharan Africa, there is no mention of follow-up being continued to determine birth outcomes after the mother and baby have been discharged. The aim of this study was to examine the specific contribution that mobile telephone calls have made to ascertain birth outcomes in a cohort study of pregnant women attending an urban health center in The Gambia. This was the first cohort study of birth outcomes in The Gambia and the first to use mobile telephone follow-up to determine the immediate birth outcome if not known, the outcome at 7 days (to measure early neonatal deaths [ENDs]), and the outcome at 28 days (to measure all neonatal deaths [NNDs]) after the delivery.

## Methods

A prospective cohort study was chosen as the study of choice as it is defined at the outset, rather than at the end of the study. At the outset, we could not anticipate the birth outcomes, but we could record the same variables for every woman. Details of the current pregnancy, obstetric history, and additional sociodemographic variables were recorded at the first visit. At the end of the study, the association between the risk factors recorded earlier in the pregnancy and birth outcomes of interest would be examined.

Ethical approval to conduct the cohort study was obtained from the combined Ethics Committee of the Medical Research Council and the Gambian Government before the commencement of the study. Informed signed consent was obtained from each woman at the first visit to the antenatal clinic after the study was explained in her own language. It was made clear that she was giving consent for her routine obstetric data to be used in a research project, as well as giving consent to an additional interview to record sociodemographic variables, and, later in the study, giving consent for mobile telephone follow-up by one of the senior midwives if a telephone call was necessary to determine the outcome of the pregnancy.

In total, 1,611 women were recruited from a total of 1,664 consecutive women attending the antenatal clinic for the first time at a small, government-supported health center in an urban area of The Gambia. Recruitment continued from the beginning of December 2012 to the end of November 2015, and follow-up continued until the final deliveries in 2016. At the first visit, we recorded whether the woman had access to a mobile telephone and noted the number. Additional numbers were also noted if available.

The outcomes of interest included the immediate birth outcome (stillbirth or livebirth), the outcome at 7 days (an END is the death of a liveborn baby within the first 7 days), and the outcome at 28 days (an NND is the death of a liveborn baby within the first 28 days). If the delivery took place at the health center immediate vital status, singleton/twin status, and birthweight were recorded in the delivery register. After delivery, every mother was advised to bring her baby to the infant welfare clinic at the health center for regular follow-up checks and immunizations.

The majority of the women and babies left the place of delivery within a few hours. Follow-up telephone calls were required if the baby was born alive at the health center, but not seen at the infant welfare clinic beyond 28 days. Follow-up telephone calls were also required if the baby was not born at the health center. In this situation, it was necessary to determine whether the pregnancy had continued, and if so, whether the baby had been stillborn or born alive. If born alive, the midwife had to determine the birthweight and whether the baby was alive 7 and 28 days after the birth. These telephone calls, potentially sensitive, were made from a quiet room in the health center by one of the senior midwives, working with the interpreter. Three attempts to contact either the mother or a relative were made.

Follow-up telephone calls were not required if the baby was born at the health center, but was stillborn, or if the baby was liveborn and was seen at the infant welfare clinic beyond 28 days.

A stillbirth was defined as a baby dying *in utero*, or during the delivery, after 28 weeks of gestation. END was defined as a live birth with death within 7 days, NND was defined as a live birth with death within 28 days, and low birthweight (LBW) was defined as a live baby being born weighing <2,500 g.

## Results

We report here, the benefit of mobile telephone follow-up. Of the 1,611 women who entered the study, access to a mobile telephone was reported by 1,511 women (93.8%) and only 100 women (6.2%) did not have access to a mobile telephone. As the study progressed, many women had access to more than one mobile telephone.

If the outcome was not known from attendance at the health center, follow-up telephone calls were conducted to determine the outcome. Despite the efforts, in some cases, it was not possible to determine any outcome at all and the outcome of 239 women (14.8%) was not known.

The immediate birth outcomes, outcomes at 7 days, and outcomes at 28 days were recorded and the additional value of the telephone calls was calculated. Although some of the stillbirths occurred at the health center, the majority occurred elsewhere and was reported during telephone follow-up. All the early and late NNDs occurred after the mother and baby had left the birth facility and were reported during a follow-up telephone call. The results at 7 days indicate the value of telephone calls to record the ENDs and those at 28 days indicate the value of the calls to record the NNDs.

### Outcome Prior to Birth or at Birth

The immediate birth outcome was known for the 968 deliveries (60.1%) that took place at the health center. Six of these were stillbirths. Of the other 643 pregnancies, the immediate outcome was determined by a mobile telephone call for a further 404 women in the cohort. These telephone calls recorded 3 late miscarriages, 1 termination, and 2 maternal deaths before delivery, as well as a further 14 stillbirths. Overall, there were 20 stillbirths and 1,325 livebirths, giving a stillbirth rate of 14.9/1,000 births after gestation of 28 weeks (95% confidence interval [CI]: 8.3–21.4).

The known outcomes at birth improved from 60.1% to 85.2% if the responses determined by telephone were included (*Table 1*).

**Table 1.** Mobile Telephone Follow-Up

OUTCOME	TELEPHONE CALL NOT REQUIRED	TELEPHONE CALL -OUTCOME KNOWN	TELEPHONE CALL -OUTCOME NOT KNOWN	TOTAL OUTCOMES KNOWN
Outcome at birth	968 (60.1%)	404 (25.1%)	239 (14.8%)	1,372 (85.2%)
Outcome at 7 days	702 (43.6%)	626 (38.9%)	283 (17.5%)	1,328 (82.5%)
Outcome at 28 days	529 (32.8%)	623 (38.7%)	459 (28.5%)	882 (71.5%)

Birth outcomes—1,611 pregnant women.

### Outcome at 7 Days

A total of 702 women gave birth at the health center and were subsequently seen at the Infant Welfare Clinic between 7 and 28 days. The remaining 909 women either gave birth at the health center, but did not return at 7 days, or gave birth elsewhere, and in both instances, a follow-up call was required. Of those, 626 women responded to a telephone call and 12 ENDs were reported. The END rate was calculated as the number of ENDs, 12 in all, divided by the total number of singleton livebirths for whom the outcome was known at 7 days. The END rate was 9.4/1,000 (95% CI: 4.1–14.7).

The overall response rate at 7 days improved from 43.6% to 82.5% with mobile telephone follow-up (*Table 1*).

### Outcome at 28 Days

A total of 529 women delivered at the health center and subsequently attended the Infant Welfare Clinic after 28 days. To determine the birth outcomes at 28 days, the women who brought their babies to the Infant Welfare Clinic at 7 days, but not beyond 28 days were included in the group, who required a follow-up call. The outcome at 28 days was not known from clinic records for 1,082 pregnancies, but, of those, 623 women were contacted by telephone and 21 NNDs were reported. The NND rate was calculated as the combined number of early and late NNDs divided by the total number of singleton livebirths with outcome known at 28 days. The NND rate was 19/1,000 (95% CI: 10.9–27.1).

The overall response rate at 28 days without the follow-up telephone calls was 32.8%, but more than doubled to 71.5% with telephone follow-up (*Table 1*).

### Birthweight

To examine the added value of mobile telephone calls to determine the birthweight, we first had to identify the live, singleton births. Twin pregnancies were excluded. There were 1,325 singleton livebirths, and of those, 948 were born at the health center, although only 933 had the birthweight recorded. Sixty-eight of the babies born at the health center with known birthweights were born weighing less than 2,500g. A further 377 live singleton births were identified from follow-up mobile telephone calls to those women who did not deliver at the health center, and a further 17 LBW babies were identified. Of the 1,252 singleton babies with known birthweight, 85 babies were born weighing less than 2,500 g (6.8%).

Of the total 1,325 live singleton births identified, the birthweight was known without requiring a telephone call for 933 babies, 70.5%. The birthweight was known for 94.6% of the live singleton births if the responses determined by telephone were included.

## Discussion

This first cohort study of birth outcomes from The Gambia is also the first cohort study to use mobile telephone follow-up to determine outcomes up to 28 days after the birth, rather than just the immediate outcome before discharge from the place of birth. The added value that a follow-up telephone call contributes has not been measured previously.

In this study, 1,611 pregnant women were enrolled in the Antenatal Clinic of an urban health center between December 2012 and November 2015 with follow-up into mid-2016. Without telephone follow-up, the immediate birth outcome was known only for those women who gave birth in the health center, 60.1% of the women. This improved to 85.2% when the telephone calls were taken into consideration. Similarly, the known outcomes at 7 days improved from 43.6% to 82.4% with telephone follow-up, and the known outcomes at 28 days improved from 32.8% to 71.5%, more than doubling the response.

It has been difficult to find any previous studies in sub-Saharan Africa using telephone follow-up to determine the health of the mother and baby following discharge after the delivery. A study from Burkina Faso, undertaken in 2011, used follow-up telephone calls to determine the health of mothers and newborns discharged with no complications from maternity units 6 h after delivery,^14^ and to determine whether there were postdischarge complications. Although it was not clear whether most of the calls were to landlines or mobiles, this study anticipated the important role of mobile telephone follow-up in the future.

There are only a very few cohort studies of birth outcomes in sub-Saharan Africa and not one of these has followed the babies beyond the birth and immediate postpartum period to record the early or late NNDs, and instead, only stillbirths, premature deliveries, and LBWs have been reported.^15–18^ The cohort study most similar to the one described here, reported from Tanzania in 2007, followed 1,688 pregnant women recruited at the antenatal clinic, who were followed to delivery,^19^ but the outcomes measured were only the immediate birth outcomes (stillbirth, intrauterine growth retardation, and LBW) before the mother had left the health facility.

By the time this cohort study commenced, mobile telephones were commonplace in urban areas of The Gambia, with 93.9% of the women having access to at least one telephone. One of our concerns at the outset was whether the responses would be truthful. In the event this did not appear to be an issue, the mothers had willingly given permission for the telephone call if necessary and, furthermore, the midwives live in the same communities as the women we were studying and often heard of a perinatal death before it was confirmed by telephone call. In addition, the calls were made by a senior midwife who had already established a good and trusting relationship with the women during their antenatal attendances.

Inevitably, there was considerable time between the initial visit and the follow-up telephone call, and during that period, we assumed that some women changed their telephone number or moved away. In the future, it would be better to check the telephone number every time the mother attends the antenatal clinic and record additional numbers where possible. As it was, in some instances, there was up to a 6-month period between noting the number and the subsequent telephone call.

Telephone calls have been shown to make an invaluable contribution in an intervention study,^8–10^ and this cohort study has shown that telephone follow-up is also an important tool in an observational study. As coverage continues to rise in sub-Saharan Africa, telephone intervention undoubtedly has an increasing role to play.
